# Principles and recommendations for incorporating estimands into clinical study protocol templates

**DOI:** 10.1186/s13063-022-06515-2

**Published:** 2022-08-19

**Authors:** Helle Lynggaard, James Bell, Christian Lösch, Amel Besseghir, Khadija Rantell, Volker Schoder, Vivian Lanius

**Affiliations:** 1 Biostatistics, Novo Nordisk A/S, Bagsværd, Denmark; 2 Elderbrook Solutions GmbH, Buckinghamshire, UK; 3 UCB Biosciences GmbH, Monheim am Rhein, Germany; 4 UCB Pharma, Brussels, France; 5 Medicines and Healthcare products Regulatory Agency, London, UK; 6 Metronomia Clinical Research GmbH, Munich, Germany; 7 Statistics & Data Insights, Bayer AG, Wuppertal, Germany

**Keywords:** Protocol writing, Protocol template, Protocol structure, Estimands, Objectives, ICH E9(R1)

## Abstract

Clinical study protocols are the foundation of good clinical studies. Prospective and multidisciplinary collaboration that pays attention to the design of all components of the study protocol can ensure that a clinical study will answer the research questions posed in a reliable manner that is meaningful for decision-makers and patients. The ICH E9(R1) addendum on estimands and sensitivity analysis in clinical trials provides a framework for clinical study planning to ensure alignment between study objectives, design, conduct, and analysis. The estimand or clinical question posed can be regarded as the backbone of the study and the clinical study protocol should reflect estimands accordingly. In practice, stakeholders are still learning how to embrace the estimand framework and how it impacts studies and study documents. In this paper, we anticipate that a protocol structure centred around estimands, or objectives rather than endpoints alone will prevail for all types of studies. To assist sponsors during this paradigm shift, this paper provides discussion and guidance for implementing the estimand framework in protocol templates.

## Introduction

In November 2019, the ICH E9(R1) addendum on estimands and sensitivity analysis in clinical trials to the guideline on statistical principles in clinical trials [1] was finalised. The addendum provides a framework for clinical study planning to ensure alignment between study objectives, design, conduct, and analysis.

In the context of clinical trials, estimands precisely describe the “treatment effect” reflecting the study objective and thus provide clarity regarding descriptions of the benefits and risks of a treatment. The need to clearly and precisely describe the question(s) of interest in a clinical study protocol (CSP), before elaborating on the study design, has already been pointed out by others [2–4], before estimand concepts were broadly introduced.

There is currently no standardised template for the format and content of a CSP. The need for such standardisation has been recognised by the International Council for Harmonisation of Technical Requirements for Pharmaceuticals for Human Use (ICH), with the creation of a new guideline, ICH M11. The ICH assembly agreed to form an Expert Working Group [5] in June 2018. The ICH M11 template, expected in 2023, will capture the estimand framework but is currently still in the development stage. Guidance for building CSP templates is provided in ICH E6(R2) [6] and SPIRIT [7]. CSP templates have been developed by research groups and organisations, including the National Institutes of Health (NIH) [8], TransCelerate [9], and individual pharmaceutical companies. Of the publicly available templates, only TransCelerate’s common protocol template (CPT) addresses the estimands framework. Individual updates of protocol templates accounting for ICH E9(R1) currently show variability in the interpretation and implementation of the estimand framework.

We are in a transition phase where stakeholders are not (yet) familiar with the estimand framework and its impacts on studies. A European Federation of Pharmaceutical Industries and Associations (EFPIA)/European Federation of Statisticians in the Pharmaceutical Industry (EFSPI) Estimands Implementation Working Group (EIWG) was established in 2019. The EIWG includes statisticians and clinicians, providing a forum for sharing industry and regulatory experiences of implementing the estimand framework in clinical studies [10]. As part of these efforts, this paper provides guidance for implementing the estimand framework in CSP templates during this transition phase.

Recommendations are presented where there is a clear benefit to a particular approach. Where there are multiple reasonable approaches, we instead present options we see as viable and allow protocol template authors to make informed choices. In some cases, the differences are just stylistic. Others are more fundamental and arise from conflicts between what is written in ICH E9(R1) and what is practically implementable in a high-quality CSP. Consequently, some options presented follow ICH E9(R1) closely but are problematic from the perspective of CSP writing, while others deviate somewhat in form from ICH E9(R1). We believe both preserve its intent and produce better protocols.

In general, remaining close to ICH E9(R1) has benefits in the short term, since the estimand concept is new, requires a change in mindset and may take a considerable time before it is fully understood and appropriately implemented. However, in the longer term, the approaches that evolve from ICH E9(R1) represent stronger ways forward.

The recommendations in this paper are those of the authors and should not be taken to represent the views of the EIWG member organisations. Template authors adopting them are responsible for ensuring that they are implemented according to existing guidelines and requirements in force at the time.

The first section provides general considerations on protocol templates, recommendations for implementing the estimands framework in CSPs and aspects to consider when choosing between one common template or multiple templates. The following section focuses on the impact of estimands on specific sections of the CSP. It discusses how and where to define estimands and the means of documenting the practical and statistical considerations for study design and conduct related to the estimands framework. The last section contains a discussion and conclusions. The Appendix provides examples.

## General considerations on protocol templates

### Estimand implementation in relation to the type of study

The principles outlined in ICH E9(R1) are relevant “whenever a treatment effect is estimated, or a hypothesis related to a treatment effect is tested, whether related to efficacy or safety” [1] and independent of the type of study (e.g. interventional randomised or observational studies), study phase, and data type (e.g. time-to-event- and longitudinal data). Consequently, they are applicable to all clinical studies for interventions such as drugs, medical devices, procedures, or vaccines.

Estimands should be defined explicitly for all study objectives that are likely to support regulatory decisions. This is widely understood as an obligation to define estimands at a minimum for confirmatory clinical studies. Regardless of regulatory requirements, clarity around what treatment effect is being estimated and what study objectives or clinical questions are being supported is beneficial in all studies. This clarity facilitates transparent and efficient communication and planning of studies as well as entire development programmes since different phase studies are interdependent. For example, the knowledge and experiences gained during phase 2 are valuable when discussing estimands and intercurrent event handling in subsequent phase 3 studies for the same drug. Therefore, the EIWG strongly encourages adopting an estimand mindset and implementing estimands in CSPs for all specific clinical questions, whether related to efficacy, safety, or benefit/risk considerations, and for all types of studies.

### Impact of estimands on protocol (template) structure

The structure of the CSP is impacted by the introduction of estimands. Traditionally, CSPs have been structured around high-level objectives and endpoints, which are usually discussed and defined early. The later descriptions of planned analyses focus on endpoints, where study endpoints are defined as response variables, i.e. characteristics of interest derived, measured, or observed on the participant level, that are chosen to assess the effect of an intervention. Note the difference in the population-level summary, that is based on aggregated data across sets of study participants.

Under the estimands framework, a clear definition of endpoints is still needed, but this emphasis on endpoints no longer fits and instead is now expanded to cover objectives and estimands. The translation of clear study objectives into key questions of interest using the estimands framework is guided by the five estimand attributes: treatment condition(s), target population, endpoint, intercurrent events and how they are addressed, and population-level summary. The endpoint is therefore one part of the estimand, which is more comprehensive and specific in describing the treatment effect that is to be assessed to address the study objective.

For CSPs using the estimand framework, it is therefore important to structure the document around the (key) clinical question(s) of interest, generally described by objectives and estimands, and link analyses to these—e.g. analyses “related to/addressing the primary objective” or analyses “for the primary estimand”. The former approach is currently reflected in a working version of the ICH M11 template, v0.6, and the latter in the TransCelerate CPT, v9.0 [9]. The defined structure should be adhered to throughout the protocol template to avoid inconsistency and confusion. Template instructions should be similarly aligned.

As a first step in the template development process, it may be helpful to address two questions:

1. Should one common template be designed to accommodate both CSPs that define estimands and CSPs that do not? (discussed in the section “One or two templates”)
2. What level of detail should be adopted when describing “clear study objectives”? (discussed in the section “Protocol template sections affected by the estimand framework”)

#### One or two templates

Typically, templates for CSPs will be used for a wide range of study types—including first-in-human studies, highly standardised food-interaction studies, dose-finding studies, pivotal studies with and without adaptive elements, and observational studies. Although the principles outlined in ICH E9(R1) are relevant “whenever a treatment effect is estimated” [1], it may appear more straightforward to apply the estimand framework to a “typical” clinical study in phase 2 or 3 than to study types not explicitly discussed by ICH E9(R1), e.g. early phase studies in healthy volunteers. Study teams may have reasons not to use the estimand framework, such as lack of experience, high standardisation of study design, or because its value is not recognised. Nevertheless, it is already clear from studies that were initiated without estimands before ICH E9(R1) was adopted, that considerations about the estimands framework can still be useful later on, e.g. in interactions with regulatory agencies or for subsequent discussions of unexpected intercurrent events such as events related to the COVID-19 pandemic [11].

As noted earlier, the introduction of estimands into a protocol template entails substantial structural changes from what is needed without them. The first step is to decide if a single template should serve all types of CSPs where estimands may or may not be described, or if separate protocol templates should be available, one for studies where estimands will be defined and a separate template for studies when they are not. Both options are discussed with their advantages and disadvantages. We recommend involving protocol template authors within a company when taking this decision.

##### Two separate templates

When using two templates, each template may focus on its relevant structure. Since there is no need to accommodate two alternative structures within a single template (e.g. through instructions, optional text, and modularity), both templates can be kept relatively simple.

This reduced template complexity comes at the expense of having to maintain two template versions and ensuring consistency in common parts unrelated to the estimands concept. The costs of maintaining two protocol templates also include the interdependencies with other document templates like the statistical analysis plan (SAP) and the clinical study report (CSR). Such interdependencies could make it necessary to manage and ensure consistency between two separate streams of document templates, with and without the estimands framework. If multiple templates are already maintained for different types of study and/or phases, then many of these costs will already be paid and this option may be quite attractive until all studies move to the estimands framework.

##### One flexible template

When using one single template for all studies (whether they use estimand concepts or not), it is recommended in the template to encourage use of estimand concepts wherever reasonable. In the instructional text of the template, a short description of “what is an estimand” could be given in addition to a statement that it is helpful to consider the estimand framework as a tool to clarify the key clinical questions to be addressed even if not mandated by regulatory requirements. In that sense, employing one template promotes the use of the estimand framework and consequently, disseminates the benefits and strengths of using it, not least transparency, across all study types. This may serve to bridge the transition to such a future when the estimand framework is used in all studies and one common template, which includes all the learnings, is used.

However, a single protocol template with specific instructions and standard text for each option can get confusing and entails a risk of instructions or section headers referring to estimands where they do not exist. Thus, where there is a shared template, estimands concepts must be included only as a combination of optional “bolt on” sections or additional details in sections that are otherwise still relevant in studies not using estimands approaches. This modularity will add considerable complexity to the template structure, including the impact on later sections and subsections, and will make templates harder to use. For sponsors currently using a single template for all studies, this may be the most reasonable approach in the short term.

##### Future directions

Four of the five estimands attributes (treatment condition(s), population, endpoint, and population-level summary measure) are relevant and required regardless of whether the estimand concept is adopted. “Intercurrent events”—even if not identified as such—as well as strategies for how to address them might partially be hidden somewhere else, e.g. in sections describing the handling of expected major protocol deviations, censoring rules, handling of treatment switching, or initiation of rescue treatment. If there is a consensus that a clear description of the treatment effect of interest with respect to these intercurrent events is valuable in all settings, then estimands concepts should be used wherever reasonable, obviating decisions about separate templates.

We anticipate that in time all studies will be based around the estimand framework, so this topic is likely only to be relevant in the short term.

*The remainder of this paper assumes that estimands concepts will be applied in all studies using a single protocol template.*

## Protocol template sections affected by the estimand framework

In this section, the impact of the implementation of the estimand framework on different protocol sections is discussed. Sections beginning with “Protocol section” in the header are referring to protocol (template) sections whereas the remaining sections are used for structuring the discussion in this paper.

### Protocol section(s) for objectives and estimands

Objectives and estimands are closely related and should be described early in the CSP. It is recommended that there is either a single section dedicated to both or two adjacent sections. Irrespective of the approach chosen, it is important that these topics are described before the study design or statistical analysis, as these are consequent to the estimand choice. Four interdependent components should be considered: objectives, clinical question(s) of interest, five estimand attributes, and rationale for the estimand.

This section should be sufficiently detailed so that all stakeholders have a clear understanding of what treatment effects are being estimated, i.e. the chosen estimands, the strategies used to handle intercurrent events, and their impacts on other parts of the protocol. However, this does not mean that every detail must be provided here as clarity is the main purpose of an estimand and excessive detail can obscure the clinical principle. Technical details that are not important for the understanding of the estimand are recommended to be described elsewhere in the protocol, in a dedicated intercurrent events section, cf. “Protocol section for intercurrent events and associated handling strategies section”. The exact split of information between the two sections may differ according to the preferences of the sponsor and, depending on the importance and complexity of the estimands, but it is important to ensure that all required information on the intercurrent events and their handling strategies is available in the protocol.

A rationale for the choice of the key estimands should be clearly stated, including a justification of the choice of intercurrent events handling strategies from the clinical/scientific perspective.

#### How to write objectives

ICH E9(R1) highlights the role of study objectives, stating that “[c]lear trial objectives should be translated into key clinical questions of interest by defining suitable estimands” and later that “[A]n estimand is a precise description of the treatment effect reflecting the clinical question posed by a given clinical trial objective” [1].

There are different ways to define objectives so that they can serve as a starting point for specifying estimands: They can be stated in great detail (e.g. detailed clinical objectives (DCOs) [12]) or in less detail (e.g. reflected in ICH E8(R1) [13]), or anywhere in between:

An example from [12] for a DCO reads like this:

The trial will compare once daily treatment with Tiotropium 5 μg + Olodaterol 5 μg fixed dose combination with Tiotropium 5 μg monotherapy in COPD patients with severe or very severe pulmonary impairment and a history of moderate to severe COPD exacerbations.

The primary trial objective is to demonstrate superiority of the fixed dose combination for the ratio of the annualised rates of moderate-to-severe COPD exacerbation over a period of 52 weeks.

The treatment effect of primary interest is while on treatment, excluding the effects of discontinuation or switching to maintenance therapies.

The same example in the format of ICH E8(R1) might be:

To compare the efficacy of Tiotropium 5 μg + Olodaterol 5 μg fixed dose combination and Tiotropium 5 μg monotherapy in COPD.

Sometimes an objective with a detail level in-between the DCO and the one suggested by ICH E8(R1) is used, and the same example might be described as follows:

To demonstrate superiority of Tiotropium 5 μg + Olodaterol 5 μg fixed dose combination vs. Tiotropium 5 μg monotherapy with respect to the annualised rates of moderate-to-severe COPD exacerbation over a period of 52 weeks in patients with a history of moderate or severe COPD exacerbations.

Less detailed objectives are common and necessitate a separate specification of the estimand(s). As an alternative, DCOs include all estimand attributes within the objective itself, including the principles for handling intercurrent events. Their focus is no longer on estimands, which are often perceived as complicated and technical by non-statisticians, but instead on “what are we trying to do?”, that is “what is the core goal of the study?” and “how do we therefore deal with the identified intercurrent events?”. Although this approach deviates from ICH E9(R1) in form, it preserves its intent, may improve interpretability and may increase engagement in cross-functional study team discussions.

According to ICH E9(R1), clinical questions of interest are more detailed translations of the study objectives, however, ICH E9(R1) neither clearly specifies their role nor discusses how they differ from objectives and estimands. It does state that intercurrent events and their strategies as well as treatment, population and endpoint should be reflected in the clinical question of interest. As such, an example could be: “What is the mean difference in *primary endpoint* after *duration* of treatment with *intervention* as compared to *placebo control* in patients with *disease* regardless of treatment discontinuation for any reason and regardless of changes in background therapy?” In this example, the clinical question of interest is basically the estimand or DCO written as a question.

If less detailed objectives are specified, a clear statement of the clinical question of interest provides the necessary context for the estimand attributes and clarifies the link between objective and estimand. Additionally, discussing that question may create more engagement from all stakeholders in the team to readily address the five estimand attributes, particularly the intercurrent events and their handling strategies. If the alternative approach of DCOs is adopted, then the clinical question is already covered effectively, so there is no need to address it separately.

The protocol (template) structure and flow of thought will therefore depend on this choice of approach to objectives. Whichever approach is adopted, this early part of the protocol should provide sufficient detail about the general purpose/aim of the study, the clinical question of interest and the corresponding estimand attributes to ensure that the design, conduct and analysis can be aligned with it.

Depending on the chosen approach there is a risk of repetition of elements belonging to the objective, the clinical question and the estimand attributes. Such repetition increases protocol length, allows inconsistencies to occur between repetitions and may discourage people from reading these important sections. These risks may be regarded as acceptable while the estimand framework is fully comprehended and implemented but approaches that minimise repetition are desirable in the long term. As the DCO format combines information from the clinical questions of interest and the estimands into the objectives, it may therefore represent an attractive way of streamlining these protocol sections once people become more familiar with the ICH E9(R1) concepts.

*In the remainder of this paper, “objective” refers to a less detailed objective as illustrated in the two examples above, while reference to a detailed clinical objective is made using “DCO”.*

#### How to write estimands

Where the DCO approach is not followed, there are various approaches to the specification of the estimand(s). Below, we give an example where two intercurrent events, “discontinuation of treatment due to any reason” and “intake of additional medication”, are handled by the treatment policy strategy. Two different forms are provided, prose and bullet points, which we consider to both be valid ways of writing the estimand. Note, we use the terminology “including the effects of” instead of “regardless of” or “irrespective of” to clarify that under treatment policy strategy, intercurrent events affect the outcomes and therefore cannot be ignored.

##### Prose

“The primary estimand (*label)* is the mean difference in change from baseline to week 26 in HbA1c between adult patients with type 2 diabetes assigned to treatment regimen X or treatment regimen Y including the effects of treatment discontinuation due to any reason and intake of additional medication.”

This form is very similar to the clinical question of interest discussed in the previous section.

Describing estimands in prose can facilitate discussion within a multi-disciplinary team during the study design stage, as well as communication with stakeholders. However, this form might lead to a description where the different attributes are not clearly discernible and writing a concise specification of the estimand in prose gets increasingly difficult as the complexity increases.

##### Bullet points

- Primary estimand (*label*):
  - ◦ Treatment condition: treatment regimen X vs treatment regimen Y including the effects of treatment discontinuation and intake of additional medication
  - ◦ Target population: adult patients with type 2 diabetes
  - ◦ Endpoint: change from baseline to week 26 in HbA1c level
  - ◦ Intercurrent events and strategies to address them: both intercurrent events (discontinuation of treatment due to any reason and intake of additional medication) are addressed in the treatment condition attribute and handled with the treatment policy strategy. Further intercurrent events are not anticipated at this time.
  - ◦ Population-level summary measure: difference in means between treatment conditions

Note, the “intercurrent events and strategies to address them” attribute differs from that in ICH E9(R1), according to which only “remaining” intercurrent events that are not addressed in the treatment condition, target population or endpoint attributes should be listed here. We consider both approaches to be valid. The key aspect is to ensure that there is clear identification of all intercurrent events and the chosen strategies, together with their rationale when documenting the estimand. For example, if the intercurrent event of death is handled by the composite variable strategy, and therefore addressed in the endpoint attribute, many stakeholders may not perceive this as an intercurrent event, but rather only as part of the endpoint definition.

The bullet points approach makes it easier to directly identify the estimand attributes. It could help ensure that all attributes are provided and all intercurrent events handled. Furthermore, it seems easier to “copy” attributes to other estimands in the same study that only differ slightly, e.g. in a single attribute, to avoid unnecessary repetition. This could for example be specified as follows:

- Secondary estimand 1 (*label*): all attributes as in primary estimand with the following difference:
  - ◦ Endpoint: change from baseline to week 26 in body weight

Alternatively, a table form can be used to show the different attributes.

Irrespective of the format used, we recommend the instructional text in the CSP template should remind protocol authors of the importance of cross-functional discussions when identifying intercurrent events and agreeing on strategies for handling them. The instructional text should also emphasise the need to consider, which estimands are relevant for each individual study rather than copying previous studies.

#### Linking objectives, estimands, the clinical question of interest, and rationale

Different suggestions of formats for providing objectives, their corresponding estimands, rationale and the clinical question of interest that can be used in the objectives section are presented in Appendix 1. The examples should not be considered exhaustive, and variations or mixtures may be considered. The advantages and disadvantages listed below will, however, be based on these examples.

An overview table, as in Appendix 1A, shows which estimands correspond to which study objective. An alternative textual format uses bullets (cf. Appendix 1B) where a hierarchy is created which presents the objective at level 1 and the clinical question of interest and estimand specification on level 2. Another alternative is a structured mixture format as used by the TransCelerate CPT v9.0 (cf. Appendix 1C), which uses a less detailed objective (cf. “How to write objectives”) in a table together with endpoints and below the table, the clinical question of interest, the five estimand attributes and the justification are provided.

A few advantages and disadvantages of these options are presented below:

All three forms are conceptually compatible with both prose and a more structured format (cf. “How to write estimands”) although the purely tabular form could pose some technical formatting challenges when used with the bullet point form as then the bullets need to be placed in a table.

A clear and integrated linking of objectives, the clinical question, estimands and the estimand rationale is important. The pure tabular format in Appendix 1A only links the objective and the estimand. The example of the format in Appendix 1B provides these links directly, whereas the TransCelerate example in Appendix 1C does not link objectives and estimands directly, but must instead provide it through referencing.

The pure tabular format seems to allow visual overview slightly better than the two other formats but seems useful only for a synopsis with just key estimands included. Many objectives and estimands could lead to very long tables that use far more document space than the bullet format would. The mixture format example in Appendix 1C has a similar advantage regarding flexibility, robustness, and space usage because only objectives and the endpoints must be accommodated for in the table part.

The DCO approach unifies the clinical question of interest, objective, and to some extent the estimand, although a section with a more detailed handling on intercurrent events is still needed elsewhere in the CSP. Overall, it produces a single concise summary that eliminates most of the linking issues.

#### Naming and referencing estimands

Labels or names could be introduced so that the estimands can be referenced in later sections without the need to repeat the complete estimand description. These labels could be generic like “primary estimand”, “secondary estimand 1”, and “secondary estimand 2” or more descriptive like “real-world effect estimand” and “pharmacological effect estimand”. However, care should be taken when naming the estimands according to the names of the ICH E9(R1) strategies as often different types of intercurrent events are handled using different strategies. Even if only one single strategy is used for all intercurrent events of a given estimand, it can create ambiguity when, e.g. the estimand is labelled “hypothetical estimand” or “composite estimand” as the hypothetical scenario or the actual composition could be different, respectively, in different studies. Within the CSP itself, using the name of a strategy may still be acceptable, because the estimand is clearly defined. However, the scope for misunderstandings increases for cross-study comparisons since names may not have been used consistently. This is especially important in the context of meta-analyses.

Similarly, the use of names associated with standard analysis practices, such as intention-to-treat and per-protocol should be avoided to prevent confusion between these approaches and estimands concepts.

### Protocol sections for study design and study conduct

The instructional text in the template should remind the CSP authors to align the design and conduct with the DCOs/estimands as they may be substantially impacted by the choices made.

A design example is, if we want to estimate the treatment effect in those who can tolerate the experimental treatment (principal stratum strategy to handle discontinuation of treatment due to adverse events), then a standard parallel-group design may not be appropriate. This is because only some patients would have taken the experimental treatment and so strong statistical assumption would be needed to handle this lack of information. Such assumptions may make estimation too unreliable for regulators to accept. However, another design could be chosen that might be more acceptable, for example in this case a randomised withdrawal design could be considered.

A conduct example is that follow-up of the patient and data collection after the occurrence of an intercurrent event is required when using the treatment policy strategy for it, so measures to ensure retention would need to be set up to collect the relevant data. The collection of detailed reasons for the occurrence of intercurrent events is required if different reasons imply different consequences for their handling [14].

The instructional text for the schedule of activities (SoA) should remind CSP authors to consider how all anticipated intercurrent events will be collected, e.g. adverse events form. The SoA should also explicitly cover monitoring of intercurrent events by requiring additional CRFs to collect relevant information that is not collected in existing ones.

### Protocol section(s) on study intervention(s) and concomitant therapy

The template instructions should guide CSP authors to clearly define the study intervention to align with the treatment condition attribute of the corresponding estimand(s). The same is true for allowed, or forbidden, additional treatments and interventions as their intake is likely to be an intercurrent event.

There should be a requirement for sufficient recording of concomitant, background, and rescue medication usage to support the intercurrent event strategies in use, e.g. type, dose, frequency, dates, or durations. This could include recording of interventions after the discontinuation of randomised treatment if such information could be helpful to address the questions of interest.

### Protocol section(s) for discontinuation of study intervention and participant withdrawal

The template should make clear the distinction between treatment discontinuation and withdrawal of study participation. The former represents an intercurrent event that needs to be considered when defining the estimand, while the latter is not an intercurrent event, but relates to missing data that should be handled within the statistical analysis.

Consequences for data collection and/or the continuation of patient visits after discontinuation of study intervention should be included in the CSP in alignment with the defined estimands.

#### Protocol section for discontinuation of study intervention

Treatment discontinuation or intake of restricted medications should not mean withdrawal from a study unless there are also safety or ethical reasons for leaving the study (as opposed to changing or discontinuing treatment). Such safety or ethical reasons ought to be rare, however, as remaining in a study ought to only require patient follow-up and consent, but not preclude necessary interventions. Such an approach should allow for data collection after intercurrent events and for subsequent use in analysis—a requirement if a treatment policy strategy has been chosen.

It is recommended to include instructional text in the protocol template aiming to collect specific reasons for treatment discontinuations, especially when strategies to address the intercurrent event treatment discontinuation depend on the reason.

#### Protocol section for participant withdrawal

The instructional text in the participant withdrawal section should not encourage investigators to withdraw participants from the study unless for safety or ethical reasons and should emphasise that treatment discontinuation does not necessarily require study withdrawal. If withdrawal from the study cannot be avoided, specific information on the reasons for it should be recorded in data to potentially identify any preceding intercurrent events that triggered the withdrawal.

### Protocol section for statistical considerations

The description in the protocol template section dedicated to the statistical considerations should focus on statistical details and methods to execute the plans described in the earlier sections.

Reference to, rather than repetition of, the appropriately described objectives, and/or labelled estimands should be made whenever possible.

Many of the necessary statistical considerations are interlinked and therefore decisions on the approach to which subsections are required and how they are related should be taken before the whole structure can be laid out. For example, there might be a subsection covering missing data handling across all analyses that could be within a “General Considerations” section or a narrower one focussing on only the primary analysis that should then be placed inside the primary analysis section.

The shift of focus from endpoints to estimands has an impact on the structure of the statistical analysis sections, too. The analysis sections should now be structured by study objectives or estimands, but not endpoints.

Routine subgroup analyses may be considered to act as a form of sensitivity analysis addressing the assumption of treatment effect homogeneity and can usually be considered to refer to the same estimand as their parent analysis. These could be placed in a general section (since they are typically performed similarly across multiple estimands) or separately in the appropriate analysis sections.

In the following sections, details on the most common statistical sections in the CSP are provided including those that are not hugely impacted by estimands.

#### Protocol section for statistical hypothesis

The recommendations regarding the specification of statistical hypotheses, confirmatory testing and controlling the type I error remain unchanged to the pre-addendum time where statistical hypotheses and multiplicity aspects are described in a separate section. It is recommended, though, to be clear about which estimand a hypothesis or a statistical test is related to.

The same considerations apply to studies that do not use frequentist testing. In general, we recommend updating this section to accommodate other types of statistical framework, e.g. Bayesian methodology.

#### Protocol section for analysis sets

ICH E9 [15] defines an analysis set as “The set of subjects whose data are to be included in the main analyses…”. ICH E9(R1) goes further, by defining the treatment effect of interest (estimand) in a way that guides both the set of participants and the relevant observations from each participant to be included in the estimation considering the occurrence of intercurrent events. Thus, a description of the selection and identification of data relevant for an analysis on a set of participants is also required.

Template authors should determine how these additional requirements should be implemented. Different proposals have been made, and each has separate implications for the CSP template:

One proposal is to amend the definition of an analysis set as used in ICH E9 with a specifier, so “participant analysis set” refers to a selection of participants and “data analysis set” a selection of data points from members of the participant analysis set. Each data analysis set would be named for later reference. The analysis sets section would therefore define two types of named sets, one for participants and one for the data points needed for estimation. This approach has been taken in the TransCelerate CPT.

Each analysis must use one data analysis set, but often the same data analysis set can be used for estimation of several estimands, typically where only the endpoint changes. Data analysis set definitions should therefore be written to try to cover as many relevant endpoints as reasonable, i.e. there should be fewer data analysis sets than estimands. It is recommended to include instructional text pointing out that the numbers of data and participant analysis sets should be minimised, and that they should be named for ease of referencing.

A second, related option is to reserve the term “analysis set” for the selection of participants and add a separate section in the statistical analysis sections of the CSP that directly links the intercurrent event handling strategies with a description of the data points required for estimation. This reduces the number of names to keep track of (as the data points sets would follow the names of their handling strategies) but may become awkward if a single handling strategy requires different estimation methods that happen to require different data selection (e.g. certain sensitivity analyses).

A third potential approach is for the data points selection to be described directly with the relevant analyses, i.e. to make data usage a property of the estimation of an estimand rather than defining standalone data sets. It is common for analyses of less important objectives to use the same estimation approaches as those for more important objectives (e.g. primary) and to refer in the CSP to the main analysis description rather than repeating it. Including data point selection in the analysis (and its description) therefore eliminates the need for separately defined data point sets.

Describing data point selection as part of the relevant analysis reduces the amount of cross-referencing and analysis set naming needed (particularly in studies with many data sets and little reuse) and may be more appropriate for time-to-event analyses (where the issue is censoring rather than data point inclusion). A drawback is this approach may make programming a little harder by not having clearly named data point sets defined in the CSP.

#### Protocol section for intercurrent events and associated handling strategies section

The template should include a section on the intercurrent events and the strategies used to address them. This could either be done on the same document level as the section for analysis sets or as a subsection of the statistical analysis section depending on how general the information intended for the section is.

The purpose of this section is to provide more detailed information on the strategies for handling intercurrent events that is not provided previously and their implementation. This may include more detailed intercurrent event definitions, expected event frequencies, technical assumptions that support statistical analyses, and potentially the associated data point selections (if not already described elsewhere, cf. “Protocol section for analysis sets”).

As the topic of intercurrent events is still quite new to clinical studies, it is also recommended that this section has instructional text reminding the authors that an overview of the frequency and timing of each type of intercurrent event by treatment group should be provided in the CSR to ease the interpretation of the estimated treatment effects.

#### Protocol section(s) for statistical analyses

Planned statistical analyses should be described for all main estimands defined in the CSP. The estimation of less important estimands (and their definition) may be deferred to the SAP.

The description of the statistical analysis methods should be structured either by study objective(s) or estimand(s), leading to a subsection “analyses for the primary (or secondary/tertiary) objective(s)” or “analyses of the primary (or secondary/tertiary) estimand(s)”. Within these sections, further structuring is recommended as illustrated by the following subsections.

##### Protocol section for missing data handling

The methodology for handling missing data should be aligned to the estimand. It can be described either in the relevant analysis section if the methodology differs across estimands or in a common separate section, e.g. in a subsection of a “General Considerations” section, if the methodology is the same across multiple estimands. The description should explicitly state the underlying assumptions to guide the relevant sensitivity analyses.

##### Protocol section for main analytical approach

The method of estimation and/or testing as well as the statistical model should be aligned with the respective estimand(s) or DCOs. Instructional text should remind authors to explicitly specify the underlying assumptions. It should be made clear which data is used in the estimation, either by naming a data analysis set, referring to the section where the data points selection is described for each estimand or, alternatively specified directly in this section (cf. Section “Protocol section for analysis sets”). The estimands themselves should have been defined earlier in the protocol and should be referred to rather than repeated in this section.

##### Protocol section for sensitivity analysis

It is recommended to include a subsection discussing sensitivity analyses for each DCO/estimand, rather than having a standalone sensitivity analysis section. This is because sensitivity analyses are directly linked to specific analyses and target the same DCO/estimand. It should be described clearly which assumption in the main analysis is being evaluated in the sensitivity analysis (e.g. missing at random assumption). Standard diagnostics should also be addressed in this section.

Sensitivity analyses are usually required for primary and key secondary DCOs/estimands to assess the robustness of the study results.

##### Protocol section for supplementary analysis

Supplementary analyses may provide additional insights into the understanding of the treatment effect related to the planned analyses described in the main and sensitivity analysis sections. ICH E9(R1) does not clearly state which estimand a supplementary analysis targets and there is currently no consensus. Compared to the main analysis, a supplementary analysis may target

1. Exclusively the same estimand
2. Exclusively different estimands (“supplementary estimands”)
3. The same or different estimands

Option 1 implies that supplementary analyses comprise competing analyses that could have been chosen as the main or potentially as a sensitivity analysis. That is, if not used as the main or to address the robustness of the results, it would be classified as a supplementary analysis.

Option 2 allows the exploration of a high-level objective from different perspectives. In this context, different estimands address different treatment effects that are closely related and address the same broadly defined study objective, i.e. their purpose is closely related to the “original” estimand. Mostly, such estimands will vary only slightly in their attributes. An example is a responder analysis of a continuous endpoint using, potentially different, cutoff values. We propose to use the term “supplementary estimands” for additional estimands that are explicitly connected to main estimands through the same high-level objective, and which are supportive in nature. As option 2 requires an explicitly different estimand is targeted, it is important to have clear terminology defining it. In addition, it brings clarity to use the term “supplementary” for both the estimand and its analysis.

Option 3 was explicitly written in the draft addendum (“Each supplementary analysis may refer to a different estimand, or a different estimator to the same estimand”) [16], but this sentence was removed from the final addendum.

The authors of this paper take any estimation of the same estimand to be sensitivity (since it is, by definition, an alternative way of estimating the same parameter) and of other estimands to be supplementary (since by definition something else is being estimated). That is, we support option 2 and believe it is the only option that provides the clarity needed to support effective implementation. We welcome further debate and publications on this topic.

Regardless of the interpretation, the protocol template should include subsections for the description of supplementary analyses supporting those objectives (or estimands) being subjected to confirmatory testing. It is not a requirement that supplementary analyses are defined for each, or indeed any (confirmatory) estimand but instructions should point out that the need for supplementary analyses should be considered.

Supplementary estimands differ from the main ones they are associated with. Depending on the strength of this association and the interpretation of supplementary analyses, the analysis of a supplementary estimand could either be described in the supplementary analysis section or as the main analysis for a standalone estimand. The former approach is preferable if the supplementary estimand is strongly related to, and less important than, the main estimand. The latter approach is more suitable when the supplementary estimand is important and of interest in its own right, for example, to address different stakeholders that have different, but important needs. This approach would require defining a standalone objective with its own estimand and separate analysis sections.

#### Protocol section for interim analysis

Depending on the use and consequence of results of interim analyses, the same principles regarding the estimand framework apply as for the final analyses. However, the instructional text should remind template users to collect robust data on the intercurrent events, since the interim analysis will depend not only on the endpoint, but also on intercurrent events that have occurred up until the time of the interim analysis. Thus, the estimand(s) to be estimated at the time of interim analysis should be clear as well as the general considerations for data integrity and type I error.

#### Protocol section for sample size determination

Sample size determination depends on the estimand(s). The instructional text in the sample size section of a protocol template should therefore highlight the need to consider and describe the expected frequency of each intercurrent event by treatment group and their consequent impact on the effect size and precision. The proportion of data that is both available and relevant for estimation should be assessed in light of the planned strategies for handling intercurrent events. For instance, treatment discontinuations may lead to a subsequent exclusion of data that is not relevant if a hypothetical approach is adopted. In contrast, this data would be included under a treatment policy approach or considered as “non-responses” under a composite approach. The effectiveness of the planned study conduct procedures for following up on patients who discontinue from randomised treatment will impact missing data assumptions for treatment policy estimation. Statistical methods used for the estimation of complex estimands may go beyond the traditional methods and it is important to align the sample size estimation to the specific analysis method. This may require simulation or additional adjustments to calculations based on simpler approaches.

Instructional text should mention that when using reference studies to derive quantitative assumptions, the estimand should be identified and differences to that in the planned study should be accounted for in the sample size calculation. Otherwise, errors may be introduced into the calculations. Likewise, when making other clinical assumptions, it is important to distinguish between expectations before and after accounting for intercurrent events—it will be necessary to adjust for the former but not for the latter.

## Discussion and conclusions

ICH E9(R1) was finalised in November 2019 and is currently being implemented by health authorities (EMA effective date: 30 July 2020; FDA publication date: 11 May 2021). We welcome the guidance, and it is clear that it has already had a positive impact on clinical studies [10]. However, the addendum does not include much guidance on implementing the estimand framework in CSPs. This paper has set out to address this, providing considerations around developing a CSP template that incorporates the estimand framework. We used our own experiences as a basis for making recommendations and have consulted a broad range of resources including the TransCelerate CPT, v9.0, a working version of the ICH M11 template from early 2021, and company-specific CSP templates.

Since the release of ICH E9(R1), members of the EIWG and the broader statistical community have raised many questions about ICH E9(R1). Some require additional clarification or help with interpretation on certain aspects from the original authors. For example, although ICH E9(R1) emphasises the importance of defining the data required for an analysis, it is not clear whether an analysis set should now be defined at the participant, or data level. In practice, both levels seem necessary, so new official terminology and definitions are probably required. It is also hotly debated whether a supplementary analysis targets a separate estimand or not, and if not, how is it different to a sensitivity analysis? It is essential to have answers to these types of questions to implement ICH E9(R1) appropriately. Otherwise, differing interpretations will take hold, people will talk at cross-purposes, and it will create difficulties and confusion when looking at studies from different sponsors.

When preparing this manuscript, we faced many challenges with implementing the ICH E9 (R1) to the letter, either because it was not clear what was required to do so, or because it would cause repetition or other issues. Some of these practical challenges resulted in us presenting different viable options that we have tried to outline in the manuscript, while in other cases, the recommendations represent compromises between competing considerations.

Further help and clarification from the original ICH E9 (R1) working group, e.g. by a revision or an official Q&A document, would be highly appreciated to ensure continued implementation of ICH E9(R1) can proceed smoothly. Papers, shared examples and presentations from industry, regulators and academia are all helpful in describing best practice, but fundamentally, they cannot change, or provide official clarification on, what ICH E9 (R1) says given its status as an official ICH guideline. We therefore believe that ongoing support by ICH is required.

The CSP is the parent of many other documents, e.g. SAP, CSR, submissions, and disclosure records. Thus, the templates for these downstream documents need to be aligned with the CSP template. Introduction of the estimands framework into study documentation must therefore begin with the CSP template, and much work has been done on this already. Further work is now needed around the specifics of introducing estimands into these other documents, such as that already proposed in the TransCelerate SAP and CSR templates [9].

As the ICH E9(R1) guidance is an addendum to ICH E9 (Statistical Principles in Clinical Trials), many stakeholders perceive estimands as a statistical topic and assume that estimands consequently belong exclusively to the statistical section of the CSP. This is a harmful misconception. Estimands are a multidisciplinary topic in drug development with an impact on study planning, study conduct, analysis, and reporting. Indeed, protocol templates are typically authored cross-functionally and therefore our recommendations in writing them are aimed at a multidisciplinary team. For all disciplines, the estimand or clinical question of interest together with the study objective should be regarded as the backbone of the study that should drive the study design, conduct and analysis and the CSP should reflect the estimand(s) accordingly.

As a first step we should be better at discussing and defining clearer objectives for studies right from the beginning of the development. Forward-looking, we expect that when the estimand topic has matured there will be less need for having separate objectives, clinical questions of interest and estimands. It should instead be possible to merge the information they contain into more precise and well-written study objectives to avoid repetition and facilitate engagement with non-statisticians, e.g. using detailed clinical objectives as proposed by [12].

Due to the complexity of the estimand framework, we recommend establishing cross-functional support groups within trial sponsors and regulators devoted to implementing the framework in clinical templates. These support groups could have a broader remit including creating awareness and education sessions but also helping teams to write estimands or objectives-centred study protocols. In addition, cross-company knowledge sharing forums like the EIWG are important in that respect to promote good practice for incorporating the estimand framework into CSPs.

In summary, the estimand framework is a novel concept within clinical development that requires a change in mindset when developing CSP templates. Our key recommendations are summarised in Table 1, but we acknowledge that we are in a transition phase and best practice is likely to mature with experience.

**Table 1:** Key recommendations

Implement the estimand framework in all studies	
Define estimands early in the CSP with a sufficient level of detail	
Keep description of intercurrent events and their strategies at a relatively high level in the objectives section to engage non-statisticians and for readability, add and refer to details in a separate section later in the CSP	
Name the estimands for ease of referencing estimands in later sections or other documents	
Describe the clinical question(s) of interest to engage non-statisticians if less detailed objectives are used	
Describe the rationale for the choice of key estimands	
Align study design and conduct with the defined estimands, including, e.g. the collection of details on intercurrent events and study intervention(s)	
Differentiate between discontinuation of treatment/intervention and study withdrawal	
Distinguish between intercurrent events and missing data	

## Data Availability

Not applicable.

## Appendix

### Structure of objectives and estimand(s) section(s)

#### Appendix 1A

The estimand description could either be in the bullet points format or in prose Tables 2 and 3.

**Table 2:** Estimand description—format 1

Objectives	Estimands
[Primary objective]	Primary estimand [estimand label] [A description of the estimand covering the five attributes: primary endpoint, target population, treatment condition(s), intercurrent events and strategies how to address them, and population-level summary measure]
	Supplementary estimand [estimand label] [A description of the estimand covering the five attributes: co-primary endpoint, target population, treatment condition(s), intercurrent events and strategies how to address them, and population-level summary measure]
[Secondary objective 1]	Secondary estimand 1 [estimand label] [A description of the estimand covering the five attributes: secondary endpoint, target population, treatment condition(s), intercurrent events and strategies how to address them, and population-level summary measure]

**Table 3:** Estimand description—format 2

Objectives	Estimands
[Primary objective]	Primary estimand [estimand label]
	• Treatment condition:…
	• Population:…
	• Endpoint:…
	• Intercurrent events and strategies how to address them:…
	• Population-level summary:…
	Supplementary estimand [estimand label]
	• Treatment condition:…
	• Population:…
	• Endpoint:…
	• Intercurrent events and strategies how to address them:…
	• Population-level summary:…
[Secondary objective 1]	Secondary estimand 1 [estimand label]
	• Treatment condition:…
	• Population:…
	• Endpoint:…
	• Intercurrent events and strategies how to address them:…
	• Population-level summary:…

#### Appendix 1B

An alternative to using a table would be to use a format which is fully based on bullet lists. One additional advantage of the bullet list is the flexibility of being able to insert even the clinical question of interest and the rationale which could be more difficult in the table format. *Italics* indicate options or optional elements.

- Primary objective: …
  - ◦ Clinical question of interest A: …
    - *▪ Primary/co-primary/multiple* estimand *(label(s))*
      - Treatment condition: …
      - Target population: …
      - Endpoint: …
      - Intercurrent events and strategies how to address them:
        - ◦ ICE 1 and handling strategy: …
        - ◦ ICE 2 and handling strategy: …
        - ◦ …
      - Population-level summary: …
    - ▪ Rationale: …
  - ◦ *Clinical question of interest B*: *…*
    - ▪* Co-primary/multiple/supplementary estimand (label(s))*
      - *description same as above*
    - ▪* Rationale: …*
- Secondary objective 1: …
  - ◦ Clinical question of interest C: *…*
    - ▪ Secondary estimand C *(label)*
      - *description same as above*
    - ▪ Rationale: …
  - ◦ *Clinical question of interest D:*
    - ▪* Secondary/supplementary estimand D (estimand label*)
      - *description same as above*
    - *▪ Rationale: …*
- *[same principle for subsequent key objectives]:* …

#### Appendix 1C TransCelerate CPT, v9.0

The TransCelerate CPT, v9.0, proposes a table linking objectives and endpoints Table 4.

**Table 4:** Objectives and endpoints table—TransCelerate CPT

Objectives	Endpoints
Primary	
•	•
Secondary	
•	•
[Tertiary/exploratory/others]	
•	•

The clinical question of interest, the estimand with its attributes, and a rationale for the estimand are to be stated below this table. Please refer to the CPT [9] for examples, which follow the structure as outlined here:

*The primary clinical question of interest for the primary objective is: What is …?*

*The estimand is described by the following attributes:*

- *Population: ….*
- *Endpoint: …*
- *Treatment condition: …*
- *Intercurrent events and strategies how to address them: …*
- *Population-level summary: …*

*Rationale for estimand: …*
